# Effect of Oligo-Fucoidan, Fucoxanthin, and L-Carnitine on Chronic Kidney Disease in Dogs: A Retrospective Study

**DOI:** 10.3390/ani14111696

**Published:** 2024-06-05

**Authors:** Naeun Hong, Ju-Hyun An, Sung-Soo Kim, Su-Min Park, Ga-Hyun Lim, Ye-In Oh, Kyoung-Won Seo, Hwa-Young Youn

**Affiliations:** 1 Laboratory of Veterinary Internal Medicine, Department of Veterinary Clinical Science, College of Veterinary Medicine, Seoul National University, Seoul 08826, Republic of Korea; naeun1001@snu.ac.kr (N.H.); ssumin94@snu.ac.kr (S.-M.P.); rkgus0724@snu.ac.kr (G.-H.L.); kwseo@snu.ac.kr (K.-W.S.); 2 Department of Veterinary Emergency and Critical Care Medicine, College of Veterinary Medicine, Kangwon National University, Chuncheon-si 24341, Republic of Korea; moonlit0816@naver.com; 3 VIP Animal Medical Center KR, Seoul 02830, Republic of Korea; ilovekh0@hanmail.net; 4 Department of Veterinary Internal Medicine, College of Veterinary Medicine, Kyungpook National University, Daegu 41566, Republic of Korea; imyein5@knu.ac.kr

**Keywords:** chronic kidney disease, reno-protective, oligo-fucoidan, fucoxanthin, L-carnitine

## Abstract

**Simple Summary:**

Chronic kidney disease (CKD) is common in old dogs and cats. Patients with CKD have impaired renal structures and decreased renal function. Because renal degeneration is generally irreversible, treatment of CKD is focused mainly on conserving the remaining renal function. In this study, we retrospectively evaluated the effects of oligo-fucoidan, fucoxanthin, and L-carnitine in canine CKD patients. The supplements were supplied for 6 months and showed a reno-protective effect, consistent with previous animal model studies. Based on our results, the combination of oligo-fucoidan, fucoxanthin, and L-carnitine has the potential to delay the progression of canine CKD and be used as an adjuvant therapy.

**Abstract:**

Chronic kidney disease (CKD) commonly occurs in old dogs and cats. Oligo-fucoidan, fucoxanthin, and L-carnitine (OFL) compounds have a variety of reno-protective properties, including anti-inflammatory, anti-oxidative, and anti-fibrotic effects. Because their effects have not been investigated in naturally occurring canine CKD, we examined their reno-protective activities in dog patients with CKD. A total of 50 patients (OFL, *n* = 28; control, *n* = 22) were included in the analysis. A significant difference was identified in serum blood urea nitrogen and creatinine concentrations between the control and OFL groups at 6 months. No significant difference in electrolytes was found between the groups. A significant difference was identified in serum creatinine concentration between the control and OFL groups in azotemic (CKD IRIS stage 2–4) at 6 months. The OFL compounds showed a reno-protective effect, consistent with previous animal studies. The OFL combination can potentially delay the progression of canine CKD and be used as an adjuvant therapy.

## 1. Introduction

Chronic kidney disease (CKD) is recognized as one of the prevalent conditions in dogs, with its occurrence ranging from 0.5% to 3.0% in the general population. In hospitalized canine populations, the prevalence can be as high as 10.0% [1]. The median survival age of dogs with CKD requires further research, but previous studies have suggested 174–336 days [2,3,4]. Because renal degeneration is generally irreversible, therapies designed for chronic kidney disease have the potential to hinder its onset, impede advancement, mitigate complications arising from decreased glomerular filtration rate (GFR), lower the likelihood of cardiovascular issues, and enhance both survival rates and overall quality of life [5]. 

Fucoidan is a sulfated polysaccharide extracted from echinoderms or marine plants such as brown algae [6]. Fucoidan has a variety of effects, including antioxidant, anti-coagulant, immunomodulatory, anti-inflammatory, and anti-tumor properties [7]. It has also demonstrated reno-protective anti-inflammatory, anti-oxidative, and anti-fibrotic effects in previous studies [6]. For example, fucoidan alleviates renal-associated blood levels in CKD animal models by reducing renal fibrosis [8]. Fucoxanthin is a carotenoid abundant in brown algae and has many pharmacological activities, including anti-inflammatory, anti-tumor, and antioxidant properties, through which it exerts renal protective effects [9,10]. L-carnitine is a quaternary amino acid involved in many metabolic pathways in the body [11]. It is obtained from dietary sources or synthesized from lysine and methionine in the liver and kidney [12]. L-carnitine alone does not reduce renal fibrosis, but L-carnitine provides synergistic effects on renal function when combined with fucoidan and fucoxanthin [13]. 

Although fucoidan, fucoxanthin, and L-carnitine (OFL) compounds have potential kidney benefits, their combined effect on naturally occurring CKD in dogs has not been investigated. In this study, we conducted a retrospective investigation into the reno-protective effects of OFL compounds by comparing kidney-related hematological values between groups of dogs that were fed OFL compounds and those that were not. The aim was to determine the potential utility of OFL compounds in canine patients with naturally occurring CKD.

## 2. Materials and Methods

### 2.1. Case Selection

This retrospective multi-center study reviewed the medical data of canine patients with CKD. The Veterinary Medical Teaching Hospital of Seoul National University and the VIP Animal Medical Center in the Republic of Korea participated in the study. The data between 1 January 2020 and 31 October 2022 were reviewed through an electronic charting program (E-friends: Pet Network Veterinarian, Seoul, Republic of Korea).

This study enrolled canine CKD patients who received and did not receive the OFL-based supplement (Fuco K, Hi-Q Marine Biotech International Ltd., Taipei, Taiwan) and had regularly visited for check-ups on health status and kidney-related blood analysis for more than 6 months. During the participation period, 45 patients received OFL compounds, but only 28 patients were monitored over 6 months. Seventeen patients were excluded for not meeting the study criteria (loss of follow-up, discontinuance of supplements, and loss of visit more than 3 times). Among the 25 patients who visited the animal hospital during the participation period who did not receive OFL compounds at the request of their owner, three patients were excluded for loss of visit more than 3 times. As a result, 50 patients (control group = 22, OFL group = 28) were enrolled in this study and monitored for 6 months.

Through e-charts, we collected patients’ information (breed, sex, and age), medical records, and concurrent disease data. We reviewed each patient’s vital signs (rectal temperature, heart rate, respiratory rate, and blood pressure), body weight, blood analysis (including serum chemistry and electrolytes), urinalysis, radiographs, and abdominal ultrasound results. The kidney value (blood urea nitrogen (BUN), creatinine (CREA), calcium, inorganic phosphate (IP), and electrolytes) was collected at least once every two month for 6 months. The diagnoses, stages, and substages of CKD were determined using the International Renal Interest Society (IRIS) criteria. 

Oligo-fucoidan, fucoxanthin, and L-carnitine were administrated using an OFL-based supplement (Fuco K). The dose of Fuco K was administered following the manufacturer’s recommendations: 1 capsule for dogs of 1–5 kg or 2 capsules for dogs of 6–10 kg, once a day, with and without food. Each capsule of Fuco K contained 125 mg of oligo-fucoidan, 125 mg of high-soluble fucoxanthin, and 50 mg of L-carnitine. 

### 2.2. Statistical Analysis

GraphPad Prism software version 6.01 (GraphPad, Inc., La Jolla, CA, USA) was used for statistical analysis. Normality was evaluated using the Shapiro–Wilk test. Results of the data are presented as the mean ± standard deviation. Differences among groups were evaluated using the Mann–Whitney test or the Kruskal–Wallis test. In all comparisons, a probability value of *p* < 0.05 was considered statistically significant unless otherwise stated. 

## 3. Results

### 3.1. Study Population

The OFL group comprised 45 dog patients (compound-receiving group). Of the 45 dogs, 4 discontinued compound usage, 7 died before 6 months (3 died from worsening CKD and 4 from other causes such as cardiogenic pulmonary edema, pneumonia, and brain tumor), and 6 were excluded due to loss of visit more than 3 times; these 17 dogs were excluded from the study. As a control group, 25 CKD patients who did not receive the OFL compounds were selected, and 3 were excluded due to loss of visit more than 3 times. A total of 50 patients were included in the analysis.

The characteristics of the patients are summarized in Table 1. Maltese, Pomeranian, Poodle, and Shih-tzu were the most common breeds in both the OFL and control groups. Between the OFL and control group, there were no statistical differences in sex, age, body weight, systolic blood pressure, and CKD IRIS stage and substages. The most common concurrent diseases were myxomatous mitral valve disease (MMVD) (*n* = 28), tracheal collapse (*n* = 22), and chronic pancreatitis (*n* = 9). Of the dogs with MMVD, some received concurrent medications, including pimobendane (*n* = 20), loop-diuretics (*n* = 17), spironolactone (*n* = 12), and Angiotensin-converting enzyme inhibitor (ACEi) (*n* = 21). Of the dogs with tracheal collapse, 15 dogs were receiving theophylline. For the management of CKD, these patients were confirmed to be taking a renal diet (*n* = 50), and subcutaneous fluid was the most common treatment (*n* = 21). Polyunsaturated fatty acids supplement (*n* = 20), renal supportive supplement (Renal Advanced, Candioli Pharma; Beinasco, Italy) (*n* = 18), and probiotics (Azodyl, Vetoquinol USA; Fort Worth, TX, USA) (*n* = 15) were the next most common. 

### 3.2. Effect of Oligo-Fucoidan, Fucoxanthin, and L-Carnitine on Renal Function in CKD Dogs

**Figure 1:**
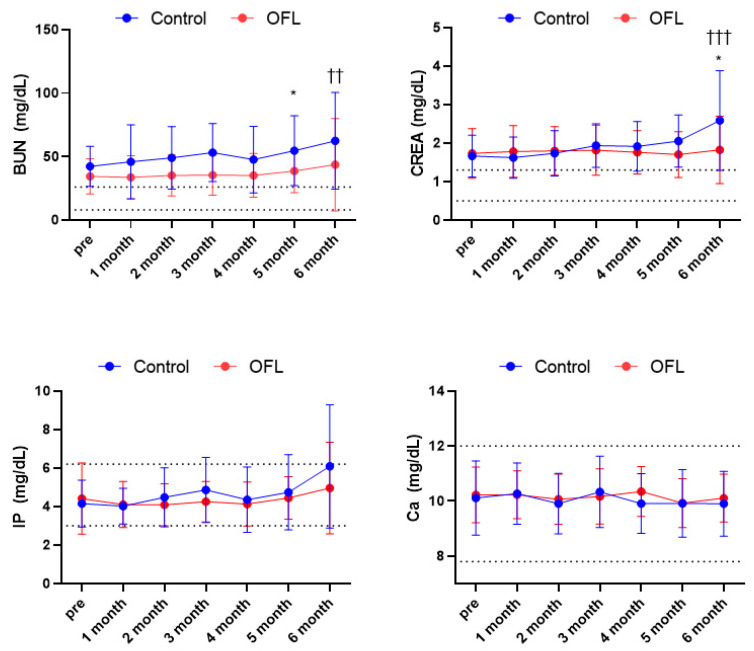
Comparison of serum BUN, CREA, IP, and Ca concentration in control and OFL group. A significant difference was identified in serum BUN concentration between the control group and OFL group at 5 months. At 6 months, in the control group, a significant increase in BUN was confirmed compared to the start of the test (*p* < 0.01), but in the OFL group, no significant increase was confirmed compared to before administration. In addition, a significant difference was identified in serum CREA concentration between each group at 6 months (*p* < 0.05). In addition, in the control group, a significant increase in CREA was confirmed compared to the start of the test (*p* < 0.001), Abbreviations: BUN, blood urea nitrogen; Ca, Calcium; CREA, creatinine; IP, inorganic phosphate. * Value on the differences between control and OFL group at 5 months and 6 months (* *p* < 0.05). ^†^ Value of the difference between the starting point of the test and the compared time point in the control group (^††^ *p* < 0.01, ^†††^ *p* < 0.001). Results are represented as mean ± standard deviation. Dashed lines indicate the reference range of serum BUN, CREA, IP, and Ca.

All of the 50 dogs in this study were evaluated for pretreatment serum BUN and CREA. At 6 months, 28 dogs in the OFL group (100%) and 22 dogs in the control group (100%) were evaluated for serum BUN and CREA levels. In the OFL group, 20 (71.4%) dogs visited the hospital every month, 3 (10.7%) dogs missed one visit, and 5 (17.8%) dogs missed two visits. In the control group, 12 (54.5%) dogs visited the hospital every month; 6 (27.2%) dogs missed one visit; 4 (18.1%) dogs missed two visits. The mean pretreatment serum BUN levels were 34.44 ± 13.90 mg/dL in the OFL group and 43.36 ± 15.76 mg/dL in the control group (reference range: 9.6–31.4). After 5 months, the BUN levels in the OFL group (38.66 ± 17.07) and control group (54.73 ± 27.59) were significantly different (*p* < 0.05). At 6 months, there was no significant difference between the two groups. However, in the control group, a significant increase in BUN was confirmed compared to the start of the test (*p* < 0.01), but in the OFL group, no significant increase was confirmed compared to before administration. The mean pretreatment serum creatinine levels were 1.66 ± 0.54 mg/dL in the OFL group and 1.73 ± 0.64 mg/dL in the control group (reference range: 0.4–1.3). Until 5 months, no significant difference in serum creatinine levels was found between the OFL and control groups (Figure 1). However, at 6 months, the mean serum creatinine levels in the OFL group (1.82 ± 0.88) and the control group (2.58 ± 1.29) were significantly different (*p* < 0.05). In addition, in the control group, a significant increase in CREA was confirmed compared to the start of the test (*p* < 0.001), but in the OFL group, no significant increase was confirmed compared to before administration. The pretreatment serum calcium levels were 10.11 ± 1.34 mg/dL in the OFL group and 10.10 ± 0.86 mg/dL in the control group (reference range: 9.0–11.9). The mean pretreatment serum IP levels were 4.41 ± 1.84 mg/dL in the OFL group and 4.15 ± 1.21 mg/dL in the control group (reference range: 2.3–6.3). In the case of IP and calcium, no differences were identified between the control group and the OFL group, and after follow-up for 6 months, there was no significant difference between periods for each group.

### 3.3. Assessment of Electrolytes after Treatment with Oligo-Fucoidan, Fucoxanthin, and L-Carnitine

**Figure 2:**
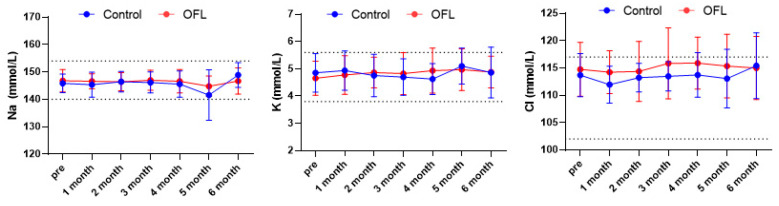
Comparison of electrolyte concentration in control and OFL group. There are no significant differences between groups. Results are represented as mean ± standard deviation. Abbreviation: Na, Sodium; K, Potassium; Cl, Chloride. Dashed lines indicate the reference range of serum Na, K, and Cl.

The serum electrolytes sodium (*n* = 47), potassium (*n* = 47), and chloride (*n* = 47) were evaluated at the pretreatment point. The mean pretreatment serum sodium levels were 146.80 ± 4.10 mmol/L in the OFL group and 145.86 ± 3.39 mmol/L in the control group (reference range: 145.1–152.6). The mean pretreatment serum potassium levels were 4.65 ± 0.62 mmol/L in the OFL group and 4.85 ± 0.70 mmol/L in the control group (reference range: 3.6–5.5). The mean pretreatment serum chloride levels were 114.74 ± 4.94 mmol/L in the OFL group and 113.67 ± 3.96 mmol/L in the control group (reference range: 113.2–122.9). No significant difference in electrolytes was found between the two groups during the study period (Figure 2).

### 3.4. Effect of Oligo-Fucoidan, Fucoxanthin, and L-Carnitine on Renal Function in CKD Dogs According to the Non-Azotemic and Azotemic Groups

Of the 28 dogs in the OFL group, 4 were non-azotemic (CKD IRIS stage 1) and 24 were azotemic (CKD IRIS Stage 2–4). Of the 22 dogs in the control group, 4 were non-azotemic (CKD IRIS stage 1) and 18 were azotemic (CKD IRIS Stage 2–4). Among the non-azotemic dogs, the differences in BUN and creatinine between the OFL and control groups were not statistically significant (Figure 2). Among the azotemic dogs, the difference in BUN between the OFL and control groups was not statistically significant; however, the difference in serum creatinine between the two groups at 6 months was statistically significant (1.95 ± 0.86 in the OFL group and 2.86 ± 1.25 in the control group) (*p* < 0.001). Interestingly, in the control group, a significant increase in BUN was confirmed compared to the start of the test (*p* < 0.001), but in the OFL group, no significant increase was confirmed compared to before administration.

### 3.5. Adverse Reactions

To assess adverse reactions to the OFL compounds, all dogs in the OFL group were reviewed for history, clinical signs, and vital signs. None of the dogs was reported to have adverse effects after administration of the OFL compounds.

## 4. Discussion

This study aimed to determine the changes in kidney-related blood factors when OFL compounds contained with oligo-fucoidan, fucoxanthin, and L-carnitine are administered in canine CKD patients and to investigate the possibility of using these compounds as an adjuvant therapy for patients with naturally occurring CKD.

According to previous studies, it has been reported that oligo-fucoidan, fucoxanthin, and L-carnitine, which we applied to patients, have the potential to have beneficial effects on patients with chronic kidney disease.

Fucoidan significantly decreased the levels of serum creatinine and urea nitrogen in a rat model of chronic renal failure [14,15]. The study claimed that the renal protective effect of fucoidan was due to its anti-inflammatory effect, and in the case of fucoidan, it was confirmed that the level of cytokines (TNF-α, IL-1β, and IL-6) was reduced in vivo, and the suppression of signal pathways (MAPK and NF-κB) was confirmed [16]. Fucoxanthin reduced apoptosis of renal tubular cells in a CKD mouse model via increasing expression of the Na^+^/H^+^ exchanger isoform 1 [9]. Furthermore, using fucoidan and fucoxanthin together has synergistic effects, including reducing serum creatinine in CKD mice, inhibiting renal fibrosis, and reducing reactive oxygen species generation and apoptosis [10,13]. L-carnitine plays an essential role in the utilization of fatty acids in the mitochondria [17]. The kidney synthesizes and metabolizes L-carnitine in animals, and a beneficial effect of L-carnitine in the kidneys has been suggested. L-carnitine inhibits gentamicin-induced apoptosis of renal tubular cells in a rat cell line via PGI2-mediated PPARα activation [18]. In CKD mice, L-carnitine treatment reduces serum creatinine levels at doses of 50 or 100 mg/kg/day [13]. In addition, the OFL combination shows greater anti-fibrosis effects on renal function in mouse CKD models [13]. However, these studies confirm the renal protective effect in an experimentally derived model of chronic kidney disease, and it is important to study what results will appear when applied to patients with actual chronic renal failure.

In this study, we investigated the results of kidney-related factors when OFL compounds were administered to dog patients with naturally occurring chronic kidney disease who visited a veterinary hospital. Comparing the changes in serum BUN and creatinine for 6 months, the control group showed a greater increase in serum BUN and creatinine than the OFL group (Figure 1). The difference between the two groups was not significant until 5 months, but statistical significance was identified at 6 months. As CKD progressed, serum BUN and creatinine levels increased in the control group. These results suggest that an OFL-based supplement could delay increases in serum BUN and creatinine levels; this effect appears after at least 6 months of administration. Although further research is needed to determine whether the experimental results and renal protection mechanisms are similar in patients who actually naturally develop chronic kidney disease, this study confirms that OFL compounds reduce the severity of kidney-related hematological values.

Research on the timing of therapeutic intervention for kidney-related treatment in patients with chronic kidney disease is still lacking in veterinary medicine. But, managing early chronic kidney disease is crucial for several reasons [19]. Firstly, early intervention can help slow down the progression of the disease, potentially preserving kidney function and preventing further damage. Additionally, addressing chronic kidney disease in its early stages helps minimize the risk of complications, such as cardiovascular issues, electrolyte imbalances, and anemia. Moreover, early management may delay the need for more aggressive interventions, such as dialysis or kidney transplantation, which are typically considered in advanced stages of the disease.

However, no research has been conducted on the effectiveness of OFL compounds according to the stage of chronic kidney disease. Through this study, the effects of OFL compounds according to the stage of chronic kidney disease are confirmed.

**Figure 3:**
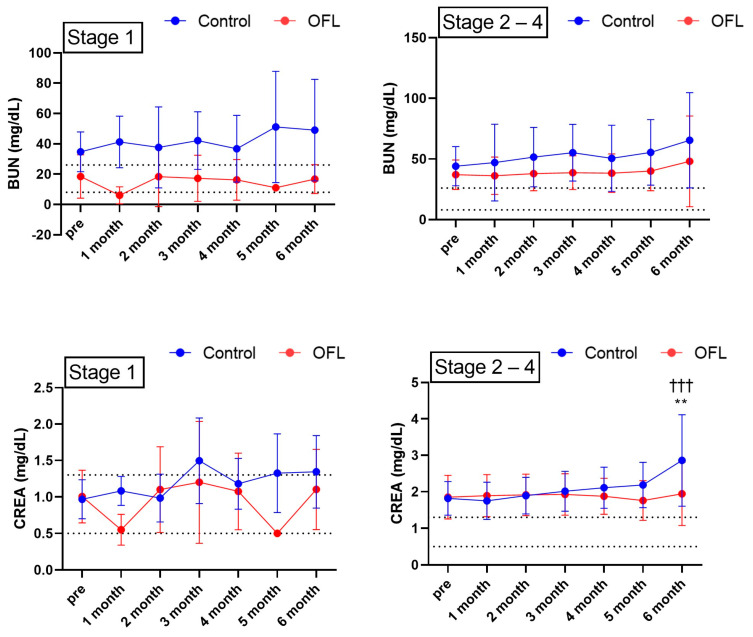
Comparison of serum BUN and CREA concentration in control and OFL group according to non-azotemic (CKD IRIS Stage 1) and azotemic (CKD IRIS stage 2−4) group. A significant difference was identified in serum CREA concentration between the control group and OFL group in azotemic (CKD IRIS stage 2−4) group at 6 months. * Value of the differences between control and OFL group at 6 months (** *p* < 0.01). ^†^ Value of the difference between the starting point of the test and the compared time point in the control group (^†††^ *p* < 0.001). Results are represented as mean ± standard deviation. Abbreviations: BUN, blood urea nitrogen; CREA, creatinine. Dashed lines indicate the reference range of serum BUN, and CREA.

When the patients were divided into azotemic (CKD IRIS stage 1) and non-azotemic (CKD IRIS stage 2–4) dogs, no significant differences in serum BUN and creatinine levels were found between the OFL and control groups in non-azotemic dogs (Figure 3). Since there were few non-azotemic dogs in both groups, the possibility of not detecting statistical differences between the groups is also considered. Therefore, it is necessary to conduct further research with larger groups. In azotemic dogs, the serum BUN of the control group was greater than that of the control group for 6 months but not statistically significant. In contrast, the change in serum creatinine levels among azotemic dogs was greater in the control group than in the OFL group, and the difference was significant at 6 months. This result suggests that the efficacy of OFL-based supplements could be confirmed more clearly in the azotemic stage of canine CKD. 

This study has some limitations. The study population was relatively small, and thus, a larger sample size is required to confirm the findings. In addition, this study measured the effect of the OFL compounds by dividing the patients into two large groups: non-azotemic (CKD IRIS stage 1) and azotemic (CKD IRIS stage 2–4). Further research is needed to evaluate the effects of the OFL compounds according to more subdivided CKD IRIS stages related to hypertension and proteinuria. Because the average age of the patients participating in the study was >10 years old, various senile diseases were common; thus, dogs taking various medications participated in the study. Therefore, additional research is needed on the possible interactive effects on patients of OFL compounds and drugs that may affect the kidneys. In addition, we could not control the precise treatment and dose differences between the groups, which may have influenced the kidney value and progression of CKD. And in this study, it was not confirmed whether OFL compounds had any effect on mortality due to the progression of CKD. The dogs participating in the test were monitored for six months after being diagnosed with chronic renal failure, but additional experiments with additional monitoring for a longer period are needed. Lastly, this study was a retrospective study, and the experimental design was not fully controlled. Monitoring the actual amount of renal supplement administered during the treatment period was not feasible. Additional closely controlled prospective studies are needed to closely evaluate the CKD efficacy of OFL compound drugs. Despite these limitations, it was found that the administration of the OFL compounds significantly decreased the BUN and creatinine levels compared to the study’s control group. This may serve as an important reference when applying OFL compounds to dogs with naturally occurring CKD.

## 5. Conclusions

In this study, we retrospectively evaluated the effects of oligo-fucoidan, fucoxanthin, and L-carnitine in canine CKD patients. The supplements were supplied for 6 months and showed a reno-protective effect, consistent with previous animal model studies. Based on our results, the combination of oligo-fucoidan, fucoxanthin, and L-carnitine has the potential to delay the progression of canine CKD and be used as an adjuvant therapy.

## Figures and Tables

**Table 1:** Characteristics of patients monitored during the 6 months participating in this trial.

Variables	Group	
	Control (*n* =22)	OFL (*n* = 28)
Breed	Maltese (3), Pomeranian (3), Poodle (1), Shih-tzu (4), Yorkshire terrier (2), Spitz (1), Cocker spaniel (1), Coton de tulear (1), Dachshund (1), Miniature pinchers (1), White terrier (1), Mongrel (3)	Maltese (8), Pomeranian (4), Poodle (5), Shih-tzu (4), Yorkshire terrier (2), Welsh corgis (1), Doberman pinscher (1), Chihuahua (1), Mongrel (2)
Sex	CM (12), M (1), SF (9), F (0)	CM (9), M (2), SF (14), F (3)
Weight (kg)	5.4 ± 3	5.4 ± 10
Age, years	14 (8–18)	13 (4–17)
Systolic blood pressure (mmHg)	150 ± 29.7	150 ± 23.8
Proteinuria (%)	Proteinuric (27.3), borderline (0), Non-proteinuric (13.6), Unknown (59.1)	Proteinuric (17.9), borderline (7.1), Non-proteinuric (35.7), Unknown (39.3)
Systemic hypertension (%)	Normotensive (27.3), Pre-hypertensive (40.9), Hypertensive (18.2), Severely hypertensive (13.6)	Normotensive (21.4), Pre-hypertensive (28.6), Hypertensive (28.6), Severely hypertensive (7.1), Unknown (14.3)
CKD IRIS stage (%)	Stage 1 (18.2), Stage 2 (77.3), Stage 3 (4), Stage 4 (0)	Stage 1 (14.3), Stage 2 (75), Stage 3 (7.1), Stage 4 (3.6)
Concurrent diseases	MMVD (17), TC (11), Hypothyroidism(1), Hyperadrenocorticism (2), Chronic pancreatitis (4), Cholelithiasis (3)	MMVD (11), TC (11), Hypothyroidism(3), Hyperadrenocorticism (2), Chronic pancreatitis (5), Cholelithiasis (4)
Treatment—for CKD	Subcutaneous fluid (8), Polyunsaturated fatty acids supplement (5), Renal supportive supplement (8), Probiotics (6)	Subcutaneous fluid (13), Polyunsaturated fatty acids supplement (15), Renal supportive supplement (10), Probiotics (9)
Treatment—for concurrent diseases	Pimobendan (12), furosemide (4), torsemide (6), spironolactone (9), ACEi (12), ARB (2), theophylline (8)	Pimobendan (8), furosemide (4), torsemide (3), spironolactone (3), ACEi (9), ARB (4), theophylline (7)

Value of ages is presented as median with range. Values of weight, systolic blood pressure are presented as means ± S.D. CM, Castrated male; F, Female; M, Male; SF, Spayed female.

## Data Availability

All data sets collected and analyzed in this study are available at the request of the corresponding authors.
